# Investigation into the Effect of Interlock Volume on SPR Strength

**DOI:** 10.3390/ma16072747

**Published:** 2023-03-29

**Authors:** Lewis Jepps, Paul Briskham, Neil Sims, Luca Susmel

**Affiliations:** 1 Department of Civil and Structural Engineering, University of Sheffield, Sheffield S1 3JD, UK; 2 Atlas Copco, Deeside CH5 2NS, UK; 3 Department of Mechanical Engineering, University of Sheffield, Sheffield S1 3JD, UK

**Keywords:** Self-Piercing Rivet, SPR, aluminium car body joining, rivet die selection, cross-section measurement, interlock measurement, Volumelock, Arealock, joint quality testing

## Abstract

During the design of automotive structures assembled using Self-Piercing Rivets (SPRs), a rivet and die combination is selected for each joint stack. To conduct extensive physical tensile testing on every joint combination to determine the range of strength achieved by each rivet–die combination, a great deal of lab technician time and substrate material are required. It is much simpler and less material-consuming to select the rivet and die solution by examining the cross sections of joints. However, the current methods of measuring cross sections by measuring the amount of mechanical interlock in a linear X–Y direction, achieved with the flared rivet tail, do not give an accurate prediction of joint strength, because they do not measure the full amount of material that must be defeated to pull the rivet tail out of the bottom sheet. The X–Y linear interlock measurement approach also makes it difficult to rapidly rank joint solutions, as it creates two values for each cross section rather than a single value. This study investigates an innovative new measurement method developed by the authors called Volumelock. The approach measures the volume of material that must be defeated to pull out the rivet. Creating a single measurement value for each rivet–die combination makes it much easier to compare different rivet and die solutions; to identify solutions that work well across a number of different stacks; to aid the grouping of stacks on one setter for low-volume line; and to select the strongest solutions for a high-volume line where only one or two different stacks are made by each setter. The joint stack results in this paper indicate that there is a good predictive relationship between the new Volumelock method and peel strength, measured by physical cross-tension testing. In this study, the Volumelock approach predicted the peel strength within a 5% error margin.

## 1. Introduction

**Figure 1:**
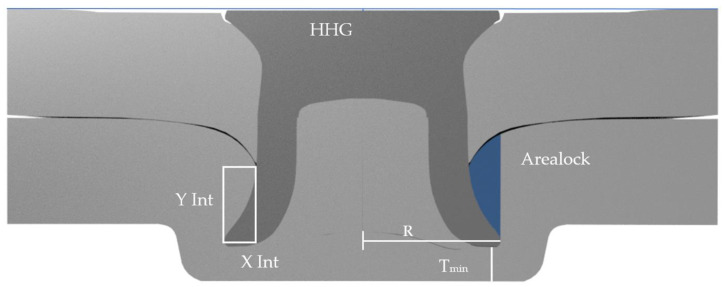
SPR cross section: Left side—conventional X–Y interlock measurement; Right side—Arealock measurement.

Self-Piercing Rivets (SPRs) are a commonly used joining technology in the automotive industry for joining both aluminium to aluminium and steel to aluminium. To select the most suitable rivet and die combination for a new joint stack, sample joints are made and physically examined. Joint characteristics such as Head Height Gap, X interlock, Y interlock, and Minimum Bottom Sheet Thickness are measured, as seen in Figure 1. These characteristics are used by automotive joining engineers as predictors of joint quality and production robustness [1].

However, the current approach of measuring X and Y interlocks does not accurately predict joint strength, as differences in the shape of the flare and amount of material above the flared rivet tip produce different cross-tension strengths, even for joints with the same X and Y interlock values. The shape of the flare is influenced by various factors, including the speed and force of the punch, die shape and material properties, and flare geometry [2,3,4].

For production, engineers must recommend a rivet and die combination for a joint stack. This often involves selecting a rivet and die combination to serve multiple material stacks on the same vehicle to reduce manufacturing complexity. The selection then must be optimised in terms of joint stack compatibility and to obtain the best possible joint strength, and, in turn, to obtain the required crash performance. Conducting physical tensile testing on every joint combination proposed for the required joint stacks is expensive in terms of both time and resources.

Therefore, it is common to narrow down the testing to the best joints from the sample group by evaluating the cross-section characteristics of the joint, which currently relies on the X and Y interlocks. However, these have been observed to be inaccurate predictors of strength, as they do not account for the full amount of material that must be defeated to pull the tail of the rivet out of the bottom sheet. The X and Y linear interlock measurement approach also makes it difficult to optimise combinations, as it creates two values for each cross section rather than a single value.

**Figure 2:**
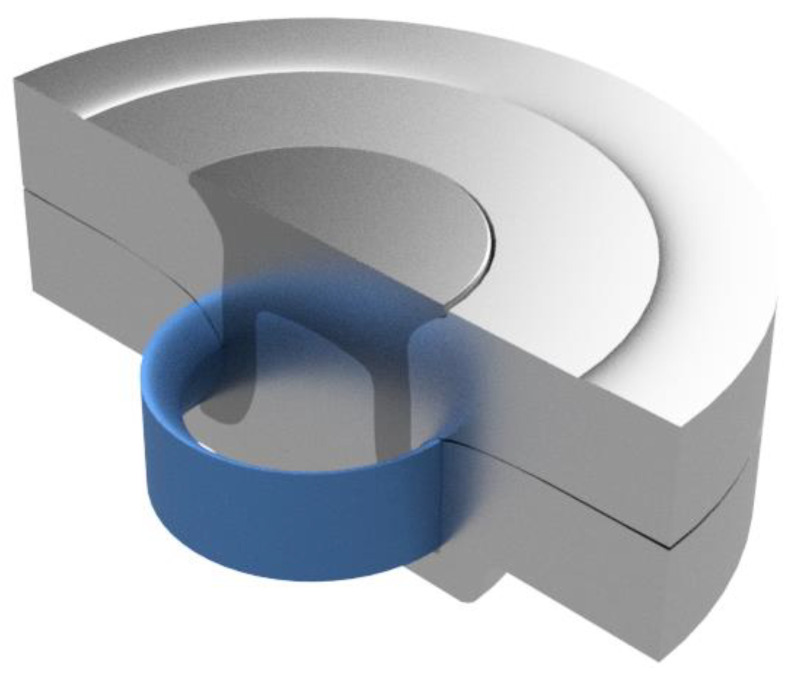
Volumelock representation—3D CAD image showing Arealock rotated through 360 degrees.

**Figure 3:**
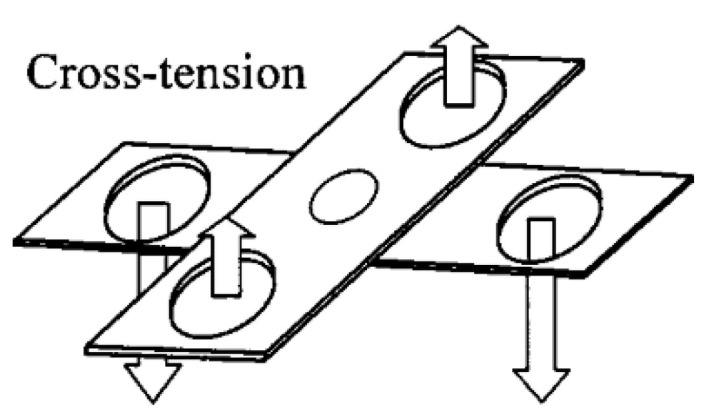
Cross-tension test configuration [5].

This study aimed to devise an improved measurement technique to optimise and select rivet and die combinations based on the physical characteristics of joint cross sections, and to investigate whether a pattern could be found to estimate the relative strength of joints with a minimal amount of tensile testing. The authors’ idea was to measure the complete area in the bottom sheet captured by the rivet flare. This area was given the name “Arealock”, and is shown in Figure 1. The Arealock is the area of material that is required to be defeated for the rivet to be pulled out of the bottom sheet. This approach was improved further by revolving the Arealock around the central axis of the rivet to give a “Volumelock” value for the complete volume of material in the bottom sheet captured by the rivet flare that must be defeated for the joint to fail from rivet pull-out. This is shown in Figure 2. This investigation focuses on the impact of Volumelock on joint strength for self-piercing riveted joint stacks in peel by analysing data from cross-tension testing, as shown in Figure 3.

Without CAD, the Volumelock can be estimated by revolving the Arealock around the central axis. This is derived from the volume of a toroid by substituting the equation for the area of a circle, which gives rise to Equation (1), where *R* is the flared rivet radius and *A* is the Arealock.

(1) V=2πRA

These new measurement techniques open up a variety of powerful measurement, analysis and optimisation techniques that will be introduced and explored in this paper.

### Current Research

Traditional interlock measurements are the only cross-sectional measurement techniques that have been applied to mechanical point joining methods such as SPR, clinching or Friction-SPR [6,7,8,9,10,11]. The state of the art tends to focus on using existing metrics to predict joint strength, with some success. However, without simulations, large errors in these predictions limit the use of these methods. Although computationally expensive, Finite-Element Analysis (FEA) is a common prediction tool in industry [12], and has shown good accuracy for quasi-static testing. Its use has grown as the models have improved, including methods such as the stress–strain history of the joint [13]. The results of Self-Piercing Rivet tests can be replicated more reliably when damage modelling is included. There are many damage models suitable for high-strain-rate deformations in SPR joint testing, such as the Johnson–Cook [14], Bonora [15] and Lemaitre models [16]. However, researchers have found that the Drucker–Prager model produced the most accurate results for aluminium top sheets [17]. The same research also used the linear X and Y interlock to predict joint strength, employing a modified shear force calculation to predict the force required to elicit material failure during cross-tension. This approach did not consider the inherent flaws in the X and Y interlock method, and therefore resulted in a 25% error in joint strength prediction.

Other researchers have employed machine learning algorithms to predict cross-section parameters such as the X and Y interlock and the resulting joint strength [18]. This method was able to predict the joint contour within 18.8% and the joint strength within 18.6%. As this method relies on the area encompassed by the joint contour, it could result in an error of up to 10%, in addition to the aforementioned 18.8% in the prediction of the joint strength. Very little previous research was found that attempted to predict the energy absorbed during failure—a key metric used by car body designers when selecting joining solutions.

Researchers have also used a neural network regression model to predict joint performance within 8.5% for steel-to-aluminium joints [19]. This required a large dataset to train and verify the model. The researchers also found the simulation of the joints predicted the maximum load fairly well; however, the model over-predicted the joint performance value, and it massively under-predicted energy absorbed due to experimental limitations in cross-tension testing, with the sheets sliding during testing. The current research in this field demonstrates that joint optimisation and strength prediction are either limited to relatively high-error methods or rely on the use of high-cost computing solutions. Due to the inherent flaws in the current measurement techniques, there exists a gap in the research for Volumelock.

## 2. Materials and Methods

### 2.1. Materials

**Figure 4:**
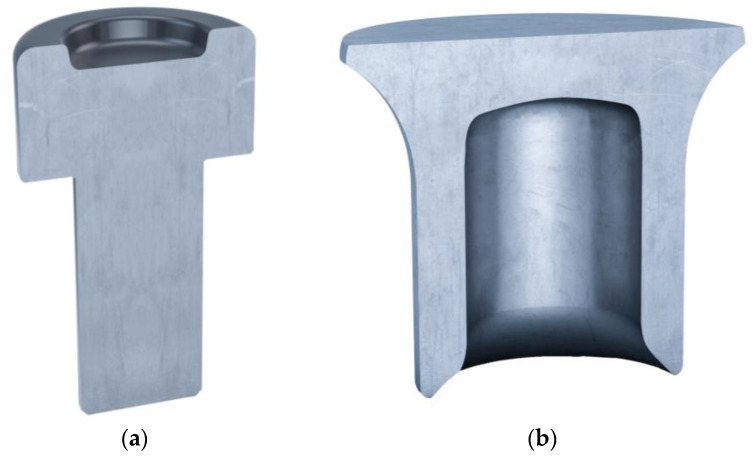
Rendered cross section images of DG10 die (**a**) and K rivet (**b**).

To investigate the effect of Volumelock, a common material stack was chosen, as shown in Table 1. The joint stack was chosen to investigate the effects of die depth and rivet length on the interlock volume. AA5754 H111 alloy was chosen because of its common use in the automotive sector for press-forming sheet panels. It is a perfect candidate for this investigation due to the fact that a huge range of rivet and die combinations can be used to join the same material stack. This means a joining engineer could find it difficult to rank the cross-sectional measurements in order to select the joints intended for tensile testing. The joint stacks were selected by preliminary investigation of the relationship between the X and Y interlock values and Volumelock. Some joint stacks had a proportional relationship between the X and Y interlocks, whilst others displayed a more complex relationship, allowing the beneficial effects of using Volumelock to be clearly visible. The die and rivet types used can be seen in Figure 4.

### 2.2. Methods

**Figure 5:**
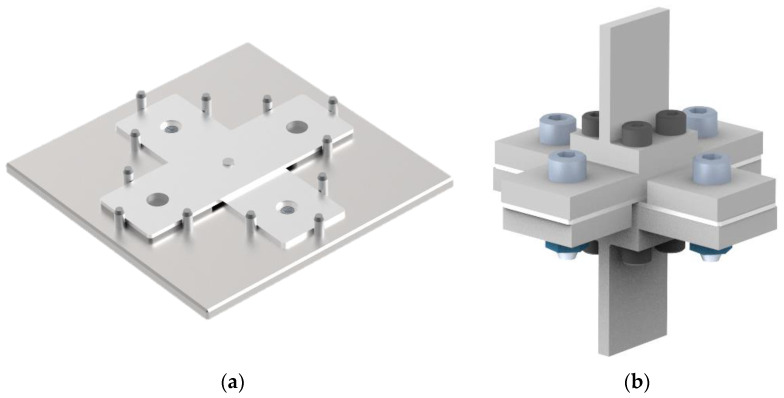
CAD models of sample creation jig (**a**) and sample testing jig (**b**).

**Figure 6:**
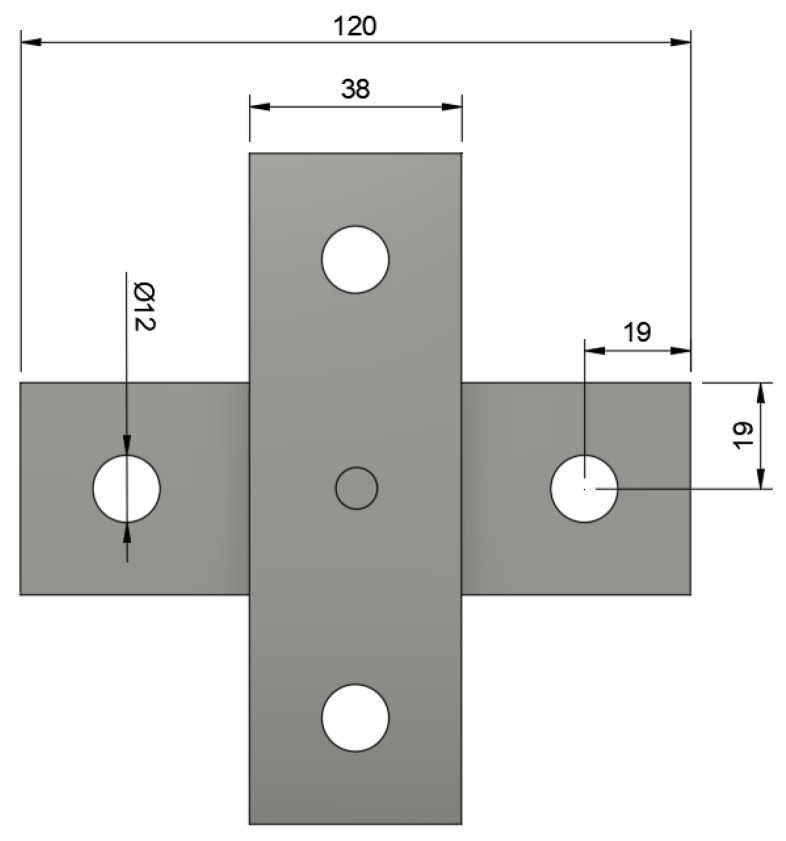
Diagram of cross-tension test specimen made from two 38 × 120 mm coupons.

**Figure 7:**
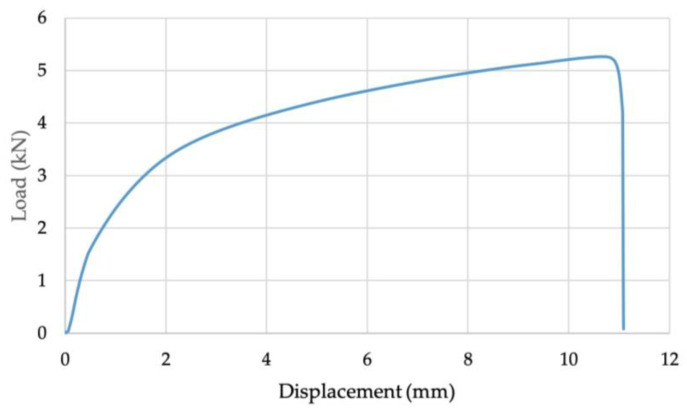
Example of cross-tension test load–displacement curve for Joint 1.

The cross-section measurement data were generated using an Atlas Copco G1.6 servo setter to make five repeats for each of the joints with the insertion parameters shown in Table 2 in 40 × 40 mm coupons. These coupons were cross-sectioned using a Struers Labatom-5 cross-section cutter (Struers, Rotherham, UK). The cut samples were then imaged using a Zeiss Stemi 508 microscope (Zeiss, Jena, Germany) with a SPOT camera to build a comprehensive set of measurement data. The X and Y interlocks were measured using rectangular measurement tools in SPOT 5.0 measurement software. These images were then exported to Autodesk Fusion 360 CAD software (Verison 2.0.15509) to measure the Volumelock directly. This was achieved by using sketch splines to capture the Arealock, as shown in Figure 1. Then, we used the revolve tool to generate a toroidal body, which was then inspected to find the volume of the shape for each side of the rivet, which was then averaged. The cross-tension samples were created using a jig, as shown in Figure 5a, from two 38 × 120 mm coupons, pictured in Figure 6. These were placed in an Instron 5892 universal test machine fitted with specially made cross-tension grips (Figure 5b). The cross-tension samples were then pulled apart at 20 mm/min. The force–displacement curves were recorded using Instron Bluehill 3 tensile testing software to yield the peak load sustained and the energy absorbed until failure, which is the area under the curve, an example of which is shown in Figure 7. All of these data were then imported to a Microsoft Excel file where the data were analysed and plotted to investigate the relationships. The mode of failure was recorded to evaluate the reason for failure (e.g., bottom sheet or top sheet pull-out). The tensile tests were repeated five times for each stack and die combination to ensure repeatability and validity, which generated an average joint performance.

## 3. Results and Discussion

**Figure 8:**
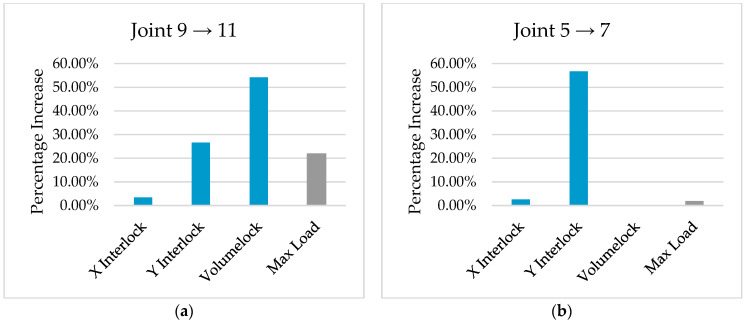
Comparative percentage increase in cross-section measurements and strength measurements for Stacks 9 and 11 (**a**) and 5 and 7 (**b**).

The X and Y interlocks were evaluated for the correlation with the maximum load during testing, and to clarify the need for a new measurement method. This was accomplished by comparing four joint stacks from the results. Two joint stacks with similar X interlocks were chosen and two joint stacks with similar max loads were chosen. The full dataset can be found in Appendix A. Figure 8 shows the percentage increase in interlock measurements and maximum load during cross-tension testing. Figure 8a demonstrates a 22% increase in maximum load with a proportional increase in the Y interlock; however, the X interlock only increased by 3%. Figure 8b demonstrates a 2% increase in maximum load with a proportional increase in X, while also showing a 57% increase in the Y interlock. These results suggest that both the X and Y interlocks independently influence joint strength; however, they do so in an unpredictable manner, making joint optimisation based on current cross-sectional measurements difficult. Figure 8 also shows the increase in Volumelock between the two joints, which tracks the increase in maximum load much more reliably, while also being a single metric. This suggests Volumelock could be a much more useful tool for engineers to optimise rivet and die combinations. Although Volumelock is inherently linked to interlock because they are both measures of the same portion of the joint, Figure 8 suggests that Volumelock is quasi-independent of the traditional interlocks.

**Figure 9:**
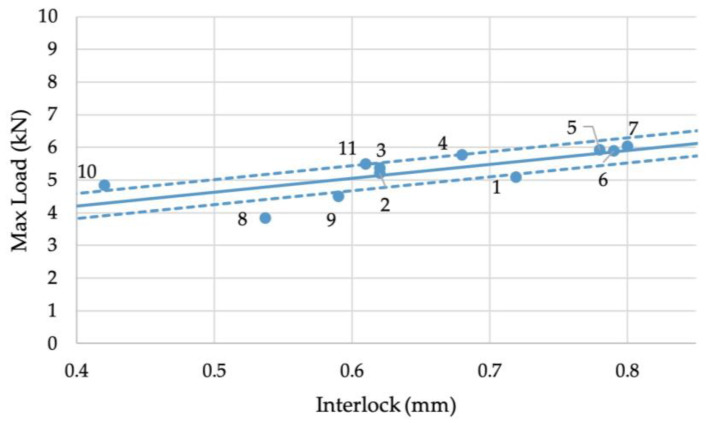
Relationship between the X interlock and maximum load during cross-tension testing.

The X and Y interlocks were plotted against the maximum load to evaluate the correlation with the regression line using the R^2^ values. The product of the X and Y interlocks, known as the interlock area, represented by the X–Y rectangle in Figure 1, was also plotted to capture the interlock in a single metric. However, this also resulted in large variations around the mean regression line, which is linear in nature due to the assumption that the pull-out strength of the joint could be calculated using the interlocks by the shear punch force, $F_{max}$, as seen in Equation (2).

(2) $$F_{max}=\tau *Y_{interlock}*\pi (D_{rivet}+{2X}_{interlock})$$

where τ is the shear stress and $D_{rivet}$ is the undeformed rivet shank diameter. The results of this calculation can be seen in Appendix A. These plots show that the R^2^ value was improved from approximately 0.55 for the X and Y interlocks to around 0.6 for the X*Y area. However, the Volumelock achieved an R^2^ value of 0.88, a significant improvement. Figure 9 shows the linear regression line of the X interlock with max load, including 95% confidence bands, accounting for the standard deviation of both the measurement and the max load. This results in a variance from the bands of ±0.38 kN if this line is used to predict the strength of joints.

The regression line intersects the Y-axis at a positive integer. This can be explained by the elastic contraction around the rivet leg, because with no interlock, the joint is still expected to have some strength due to material springback, much like a nail in wood. However, a larger dataset must be used to investigate the intersection of the axis with lower interlocks.

**Figure 10:**
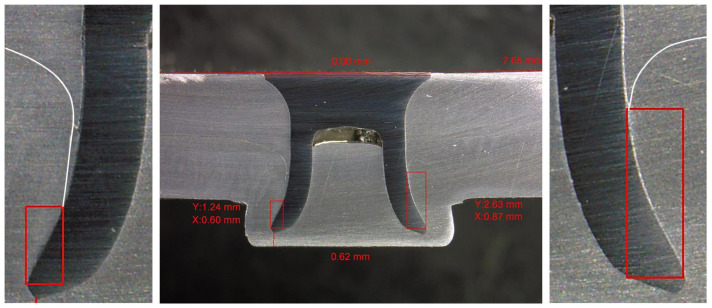
Cross-section image of Joint 7 showing effect of top sheet drag down on X–Y interlock.

**Figure 11:**
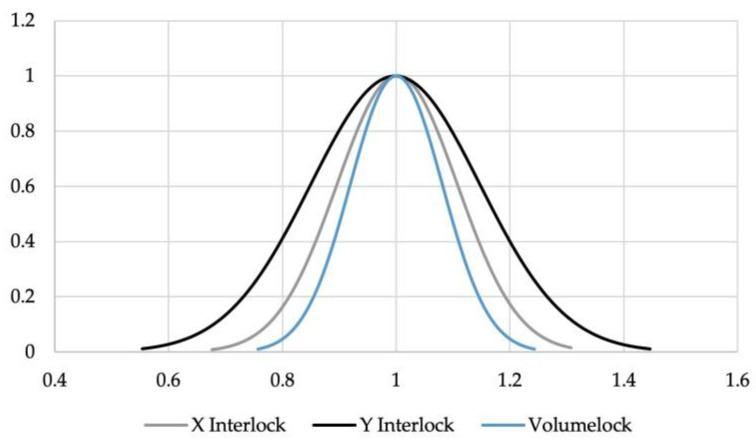
Normalised mean bell curves for measurement variations.

The X interlock also presented other issues, mainly with top sheet pulldown. When this occurred, the top sheet material was drawn down to the tail of the rivet, artificially reducing the interlock, as seen in Figure 10. Examples of the other joint cross sections can be seen in Appendix B. This is an interesting phenomenon, as the right hand of the joint had a Y interlock more than double that on the left-hand side, and an X interlock 45% greater than that on the left-hand side. Despite the much larger interlock on the right-hand side, the left-hand side actually resulted in a 24.5% greater Volumelock, indicating that Volumelock is a more useful way of assessing the joint strength by measuring a cross-section image. Another issue with traditional interlock measurement methods is the variation of measurements across repeats of the same joint. The normalised bell curves for the variation of the measurements of the interlocks and Volumelock are shown in Figure 11. This demonstrates a lower variation in the measurements for Volumelock than traditional interlocks.

**Figure 12:**
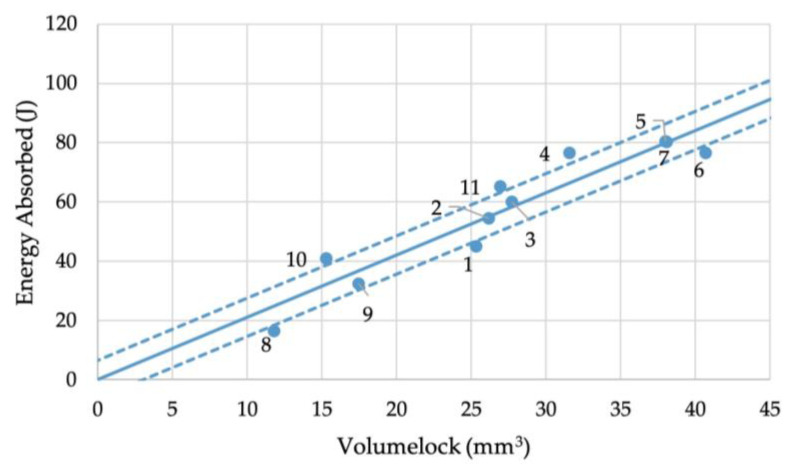
Relationship between Volumelock and energy absorption during cross-tension testing.

This then creates a need for the measurement techniques introduced by the authors above. By measuring the Arealock of the joint cross section and revolving this to give the Volumelock value in the bottom sheet, then comparing this to the energy absorbed during cross-section testing, we can derive the relationship seen in Figure 12.

In the figure, the X-axis is volume and the Y-axis is energy absorbed. From this we can derive the gradient as the energy absorbed per unit volume, which is directly proportional to the energy absorbed per unit mass, or specific energy absorption (SEA). This is a material and geometry constant often used to quantify joint failure performance, particularly in crash testing, resulting in a linear regression line. For this joint stack, the gradient was 2.1 J/mm^3^, or a specific energy absorption of 774.32 J/g. The volume captured by the rivet head in the top sheet remained relatively consistent across the range of lower Volumelocks, allowing a fair comparison to be drawn between them. The regression line intersects the axis at (0,0) because the gradient is the specific energy absorption, meaning zero mass is unable to absorb energy. Further work should be conducted to understand the relationship at higher Volumelock values when the failure mode changes, as this study only focused on the failure mode of tail pull-out.

Although energy absorbed during failure is a critical metric, another useful metric is the maximum load the joint can withstand before failure. As seen in this research, and in research by others, in different joint stacks resulting in different interlocks [20], a linear relationship can be drawn between the energy absorbed and the maximum load the joint is capable of carrying for a given joint stack. This means that we can apply the same relationships found with the energy absorbed to the max load. This method can also be applied to similar processes, such as Friction Self-Piercing Riveting (FSPR), with research replicating these relationships with differing tensile tests [21]. However, this also resulted in a lower SEA of 461 J/g and a zero Volumelock energy absorption of non-zero due to the solid-state bonding achieved in the process, indicating that further work should be conducted to investigate this effect alongside hybrid bonding with adhesive.

**Figure 13:**
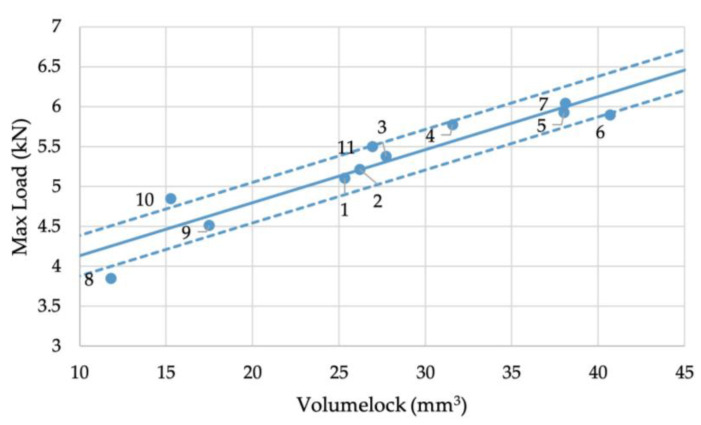
Relationship between Volumelock and maximum load during cross-tension testing.

As with the energy absorbed, there is variation around the regression line. This is due not only to experimental and material variations, but also to variations in the upper sheet Volumelock. As mentioned previously, this is not large. However, a mean of 71.4 mm^3^ and a standard deviation of 4.82 does result in noticeable variations. The data points and regression line can be seen in Figure 13. The data points fit well with the regression line, showing that the max load can be predicted from Volumelock with a 95% certainty that the prediction will be within ±0.26 kN, or within 5% of the mean strength from the dataset in this study.

**Figure 14:**
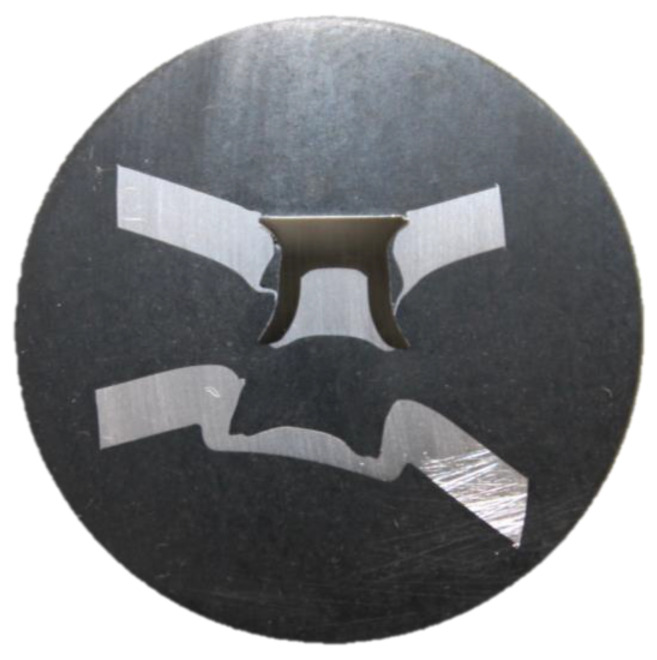
Image of the tail pull-out failure mode experienced in this study.

As this is a new technique, we suggest many avenues for exploration. Further work should be conducted to understand the relationships at the upper and lower bounds of the investigated dataset. In the current results, only one failure mode was observed, that is, tail pull-out, which is pictured in Figure 14. This is likely as a result of the Volumelock captured by the rivet head in the top sheet being greater than that in the bottom sheet, which means increased resistance to failure. This opens up the possibility for further work to predict the failure mode from the upper and lower Volumelocks, which would be a novel prediction tool. One suggestion for further use of this technique would be to rank joints absolutely. This may be achieved by dividing the lower Volumelock by a critical Volumelock, i.e., the volume at which the failure mode switches to head pull-through and therefore reaches a maximum strength, allowing joints to be assigned a percentage rank or score out of 10. This could result in a few cross sections being made, or even simulated, and the strongest joint stack could be selected without a single strength test being conducted. 

Another powerful use of this metric could be to predict the Volumelock and therefore the strength of the joint, as the joint is made via the force–displacement curve on the setting machine, which is similar to previous research which used it to predict the X interlock [22]. This could then be implemented as a non-destructive monitoring method during production to ensure that every joint made meets the strength requirements.

## 4. Conclusions

In this study, the authors developed and tested a new cross-section measurement technique intended to aid joining engineers tasked with selecting the best-performing rivet and die combination for a set of SPR joints.

- This study resulted in a new measurement method for cross-section analysis that is potentially capable of predicting tensile test joint strength with enough accuracy to remove the need for conducting extensive physical tensile testing.
- The measurement technique represents a new way of optimising joint parameter choice through a single measurement, improving on current measurement and prediction techniques in terms of accuracy and precision.
- The relationships between joint performance and Volumelock measurement were investigated and found to be a function of specific energy absorption, which in turn is a function of the material and geometry constants of the tested samples. This opens up the possibility for future work to calculate values useful to car body designers and joining engineers without the need for extensive physical strength testing.
- Further work should be conducted to fully understand the effect of geometry and material on the relationship between Volumelock and joint strength.
- In this initial work we have only begun to explore what might be achieved using this new approach, and we encourage other researchers to help us further develop this interesting new method for the wider benefit of the joining community.

## Figures and Tables

**Table 1:** Joint stack chosen for the investigation.

	Top Sheet		Bottom Sheet	
	Alloy	Thickness (mm)	Alloy	Thickness (mm)
Stack 1	AA5754 H111	3.0	AA5754 H111	3.0

**Table 2:** Stack 1 insertion parameters.

Test ID	Rivet Type	Rivet Length (mm)	DG Die Cavity (Diameter )	DG Die Cavity (Depth)	Insertion Force (kN)	Insertion Velocity (mm/s)
1	K50A42AH00	8.5	10	100	70.96	340
2	K50A42AH00	8.5	10	120	72.40	340
3	K50A42AH00	8.5	10	140	73.80	340
4	K50A42AH00	8.5	10	160	72.28	330
5	K50A42AH00	8.5	10	180	64.14	300
6	K50A42AH00	8.5	10	200	53.62	270
7	K50A42AH00	8.5	10	220	50.30	260
8	C50D42AH00	6.5	10	200	46.7	230
9	K50742AH00	7.0	10	200	50.66	250
10	K50M42AH00	7.5	10	200	52.38	260
11	K50842AH00	8.0	10	200	53.80	270

**Figure A1:**
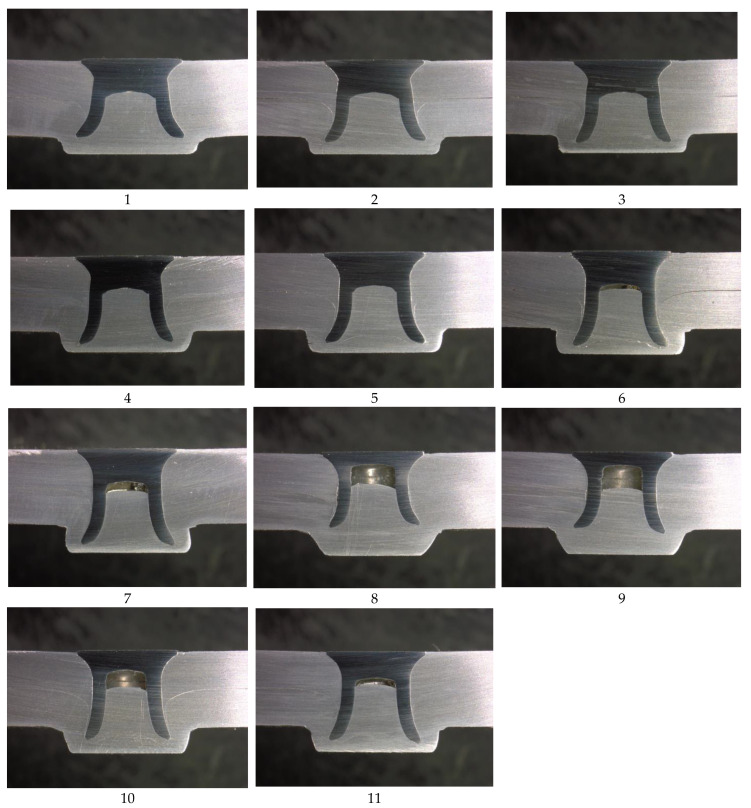
Experimental joint cross sections. (Joint ID **1**–**11**).

## Data Availability

Data are contained within the article.

## Appendix A

**Table A1:** Experimental results.

Test ID	Rivet	Nominal Rivet Length (mm)	Die	Die Depth (mm)	Avg X Interlock (mm)	Standard Deviation X interlock	Avg Y Interlock (mm)	Standard Deviation Y Interlock	Avg Volumelock (mm^3^)	Standard Deviation Volumelock	Avg Max Load (kN)	Standard Deviation Max Load	Avg Total Energy Absorbed (J)	Standard Deviation Energy Absorbed	Calculated Shear Punch Force (kN)
1	K50A42AH00	8.5	DG10-100	1	0.719	0.0572	1.11	0.079	25.3	1.80	5.10	0.131	44.9	2.29	3.74
2	K50A42AH00	8.5	DG10-120	1.2	0.620	0.0560	1.27	0.029	26.2	2.11	5.21	0.115	54.4	2.66	4.17
3	K50A42AH00	8.5	DG10-140	1.4	0.620	0.0231	1.33	0.178	27.7	1.30	5.37	0.109	60.0	2.62	4.37
4	C50D42AH00	8.5	DG10-160	1.6	0.680	0.0862	1.47	0.211	31.6	3.45	5.77	0.241	76.5	6.07	4.92
5	K50M42AH00	8.5	DG10-180	1.8	0.780	0.0578	1.57	0.301	38.0	2.19	5.93	0.241	80.5	3.98	5.41
6	K50742AH00	8.5	DG10-200	2	0.790	0.1467	1.95	0.640	40.7	4.47	5.89	0.136	76.6	3.26	6.74
7	K50842AH00	8.5	DG10-220	2.2	0.800	0.0374	2.46	0.283	38.1	1.10	6.04	0.109	80.2	3.34	8.53
8	K50A42AH00	6.5	DG10-200	2	0.537	0.0612	1.07	0.119	11.8	1.15	3.85	0.079	16.3	0.61	3.44
9	K50A42AH00	7	DG10-200	2	0.590	0.0762	1.24	0.187	17.5	1.67	4.51	0.069	32.4	0.75	4.04
10	K50A42AH00	7.5	DG10-200	2	0.420	0.0967	1.22	0.172	15.3	3.20	4.85	0.179	40.9	3.87	3.76
11	K50A42AH00	8	DG10-200	2	0.610	0.0581	1.57	0.224	27.0	1.78	5.50	0.168	65.2	0.86	5.14
